# OceanRAIN, a new in-situ shipboard global ocean surface-reference dataset of all water cycle components

**DOI:** 10.1038/sdata.2018.122

**Published:** 2018-07-03

**Authors:** Christian Klepp, Simon Michel, Alain Protat, Jörg Burdanowitz, Nicole Albern, Marvin Kähnert, Andrea Dahl, Valentin Louf, Stephan Bakan, Stefan A. Buehler

**Affiliations:** 1 Cluster of Excellence CliSAP (Climate System Analysis and Prediction) and Center for Earth System Research and Sustainability (CEN), Initiative Pro Klima, Universität Hamburg, 20146 Hamburg, Germany; 2 Meteorological Institute/CEN, Universität Hamburg, 20146 Hamburg, Germany; 3 Max Planck Institute for Meteorology, 20146 Hamburg, Germany; 4 Bureau of Meteorology, VIC 3008 Melbourne, Australia; 5 Eigenbrodt GmbH & Co. KG, 21255 Königsmoor, Germany; 6 Monash University, VIC 3008 Melbourne, Australia

**Keywords:** Ocean sciences, Atmospheric science, Hydrology

## Abstract

OceanRAIN—the Ocean Rainfall And Ice-phase precipitation measurement Network—provides in-situ along-track shipboard data of precipitation, evaporation and the resulting freshwater flux at 1-min resolution over the global oceans from June 2010 to April 2017. More than 6.83 million minutes with 75 parameters from 8 ships cover all routinely measured atmospheric and oceanographic state variables along with those required to derive the turbulent heat fluxes. The precipitation parameter is based on measurements of the optical disdrometer ODM470 specifically designed for all-weather shipboard operations. The rain, snow and mixed-phase precipitation occurrence, intensity and accumulation are derived from particle size distributions. Additionally, microphysical parameters and radar-related parameters are provided. Addressing the need for high-quality in-situ precipitation data over the global oceans, OceanRAIN-1.0 is the first comprehensive along-track in-situ water cycle surface reference dataset for satellite product validation and retrieval calibration of the GPM (Global Precipitation Measurement) era, to improve the representation of precipitation and air-sea interactions in re-analyses and models, and to improve understanding of water cycle processes over the global oceans.

## Background & Summary

Global ocean water cycle monitoring is essential for a successful understanding of the climate system^1–4^. The net gain (precipitation) or loss (evaporation) of water through the ocean surface yields the freshwater flux, linking the global water cycle to the energy budget through latent heat exchange^5^. The freshwater flux couples the ocean to the atmosphere, driving oceanic and atmospheric circulations with impacts on sea surface temperature and salinity^6,7^. Several factors complicate the measurement of precipitation such as its intermittent nature, inhomogeneous spatial distribution, intensity variations and phase changes, as well as technical detection limits for very light precipitation. Despite its large uncertainties^8,9^, precipitation is likely the single most essential climate variable (ECV) to characterize^4,3,10^.

Recent satellite remote sensing products provide unprecedented spatio-temporal coverage of evaporation, fostered by the SeaFlux community^11^ under the auspices of the World Climate Research Programme (WCRP), and precipitation through the International Precipitation Working Group^9,12,13^ (IPWG), co-sponsored by the World Meteorological Organization (WMO). The TRMM^14^ (Tropical Rainfall Measurement Mission), CloudSat^15,16^ and Global Precipitation Measurement^17^ (GPM) satellite missions, aided by their spaceborne precipitation radars TRMM-PR, CloudSat-CPR and GPM-DPR boosted the emergence of precipitation products^18–24^. The Integrated Multi-satellitE Retrieval for GPM^25^ (IMERG) retrievals discriminate between rain and snow and feature increased sensitivity to light rain. However, all these products exhibit sensitivity issues as well as large differences among each other, especially for light precipitation and high-latitude cold-season precipitation^26–29^.

Therefore, thorough evaluation of the associated errors, biases and uncertainties to improve the satellite retrievals requires a comprehensive and consistently derived in-situ surface-reference dataset over the global oceans with special emphasis on the precipitation parameter^4,30^.

The hitherto lack of such a validation dataset mainly arose from the unavailability of suitable instrumentation. Gauge-type devices remained the principal source of in-situ precipitation measurement over ocean on ships^8,31–33^ and buoy networks^34–36^. They serve as an input to a variety of ship-based precipitation estimates^31,37–41^. However, gauges are generally not well suited to this task because of large wind-induced rainfall undercatch resulting from superimposed ship’s speed, surface wind speed and turbulent flow distortion around the ship superstructure^42–45^. Moreover, snow and mixed-phase precipitation is either blown over or clog the gauge orifice resulting in false measurements.

In addition to the rain rate provided by gauges, particle size distributions (PSDs) are required to derive microphysical rain and snowfall properties and reflectivities at different radar operating frequencies. These parameters are essential to accurately convert the radar reflectivity into a precipitation rate^46,47^. Such in-situ validation data for retrieval calibration^48–50^ can only be provided by disdrometers^8,30,51,52^. However, most of the existing disdrometers are not designed for all-weather shipboard operation and thus do not meet the requirements for strong turbulence, frequently varying wind directions and sea state.

Therefore, this observational gap in high-quality precipitation measurements over the ocean^51,53,54^ remained critical for meeting the requirements of validating an ECV^30^. This motivated us to develop and implement a monitoring network for in-situ precipitation, evaporation and freshwater flux to support the requirements of the international science teams of IPWG, GPM-GV (Ground Validation), SeaFlux and OceanObs^11,13,17,55^ and the Global Climate Observing System^4^. All satellite-based precipitation retrievals and products as well as re-analyses and models involving air-sea interaction would benefit from such new in-situ reference dataset^8^.

To bridge this important information gap, we present the surface reference dataset OceanRAIN-1.0 (Ocean Rainfall And Ice-phase precipitation measurement Network) (Data Citation 1, Data Citation 2 and Data Citation 3). OceanRAIN is the first in-situ global ocean shipboard dataset comprising 75 meteorological and oceanographic parameters including consistently derived along-track precipitation, evaporation, the resulting freshwater flux as well as surface turbulent fluxes. The precipitation parameters include rain, snow and mixed-phase precipitation occurrence, intensity and accumulation, all derived through PSDs based on automated ODM470 optical disdrometers that were specifically designed to meet all-weather shipboard requirements. All relevant microphysical precipitation parameters and reflectivities at different radar frequencies are also provided. These datasets are collected during the ongoing long-term installations and special campaigns onboard eight research vessels from June 2010 to April 2017 covering all latitudes, oceanic basins and seasons and comprise more than 6.83 million minutes including 696,740 precipitation minutes (Table 1). OceanRAIN-1.0 data is publicly available through the website http://www.oceanrain.org/ and the World Data Center for Climate (WDCC).

## Methods

### Experimental design

OceanRAIN is designed to meet the IPWG, GPM Ground Validation, SeaFlux and OceanObs requirements for high-quality in-situ water cycle reference data of precipitation, evaporation and freshwater flux measurement over the global oceans. OceanRAIN is primarily intended for validating satellite data products and retrievals and for understanding and reducing their uncertainties. The Global Climate Observing System^4^ calls for the development and implementation of improved methods for observing precipitation, and deriving associated products. It also strongly recommends assessing uncertainties of satellite precipitation products, using newly developed automated in-situ data over the global oceans and with special focus on high-latitudes. This demand is also supported by the recommendation of the Coordination Group for Meteorological Satellites (CGMS) to maintain existing and explore new sources of in-situ precipitation measurement in data sparse regions such as the global oceans.

Accurately simulating precipitation processes and associated statistical rainfall properties (accumulation, instantaneous intensity and frequency of rainfall) with general circulation models is paramount for hydrological applications, numerical weather prediction and climate change studies. As discussed in Stephens *et al*.^56^, changes to both the frequency and intensity of precipitation occur in climate-warming experiments. The evaluation of rainfall accumulation over seasonal or longer time-scales provides limited insights into the validity of convective processes simulated by models. Therefore, several studies have attempted to evaluate both the intensity and frequency of rainfall simulated by large-scale models, using either land-based observations^57,58^ or oceanic rainfall derived from satellite measurements^56^. These comparisons revealed that, although modelled rainfall accumulations at different temporal scales are generally reasonably accurate, general circulation models tend to overestimate the frequency and underestimate the intensity of precipitation, with different behavior of the models in different regions and latitudes^56^.

Oceans cover about 71% of Earth’s surface and accounts for an estimated 78% of global precipitation^36^. The hitherto lack of surface precipitation data^36,51^ hindered validation efforts because of the unavailability of a capable instrumentation, e.g. disdrometers, for shipboard operation. This clearly highlights the need for global ocean observations of precipitation in different large-scale regimes to better understand underlying causes for model discrepancies and to help improve model parameterizations of precipitation processes.

Reliable in-situ observations of rain, snow and mixed-phase, occurrence, intensity and accumulation through PSDs over all oceanic basins and seasons are critical to validate the precipitation ECV. Since 2009, the OceanRAIN project tackled the challenge involved to develop, operate and long-term maintain a network of automated disdrometer systems under all-weather conditions onboard globally operating Research Vessels (RVs) and to develop an automated data post-processing chain^30^.

Consequently, the optical disdrometer ODM470 initially developed by GEOMAR^59^ in Kiel, Germany, was selected because it was designed to meet all-weather shipboard requirements. Within OceanRAIN we tested, utilized and further developed the disdrometer into a fully automated measurement system, the ODM470 (ref. 30) built by the company Eigenbrodt GmbH & Co. KG in Königsmoor, Germany. During its development and testing period the ODM470 was already successfully used in several studies and shipboard campaigns including first validation of satellite data and the evaluation of reanalysis products^60–64^.

To complement the in-situ shipboard precipitation measurement, OceanRAIN also ingests and stores all routinely measured shipboard meteorological and oceanographic data streams needed to derive the evaporation parameter. With the inclusion of validated true-zero precipitation measurements, OceanRAIN is to date the only publicly available comprehensive dataset providing the full suite of water cycle parameters, including the along-track freshwater flux in a consistent framework at 1-min resolution. RVs with long-term OceanRAIN installations offer a perfect framework because they operate in all climate-relevant areas and seasons over the global remote oceans including the high-latitudes and do not circumvent routinely high-impact weather. This largely avoids the so-called fair weather bias that occurs when merchant ships or cruise liners circumvent high-impact weather along their main shipping routes. For these reasons the OceanRAIN data set comprises the entire spectrum of weather events including extreme values in remote locations.

These long-term installations are complemented by RVs providing short-term campaign data with special emphasis on water cycle analysis. We contributed to the tropical Pacific SPURS (Salinity Processes in the Upper Ocean Regional Study) campaign onboard RV Roger Revelle, and the CAPRICORN (Clouds, Aerosols, Precipitation Radiation and atmospherIc Composition Over the southeRN ocean) cruises onboard RV Investigator with a focus on GPM and CloudSat satellite validation using collocated ship underpasses (see Technical Validation section). Table 1 lists the eight ships in the OceanRAIN-1.0 fleet. All large German RVs are long-term equipped with the OceanRAIN instrumentation covering the Atlantic Ocean and also parts of the Pacific Ocean including the high latitudes and cold seasons. RV Polarstern, RV Investigator and MS The World focus on the high-latitudes with a large precipitation fraction falling as mixed-phase and snow at temperatures as low as −30 °C.

Beyond the application of satellite product validation and retrieval calibration, the OceanRAIN weather and ocean monitoring is used to develop methods to improve point-to-area representativeness analysis of precipitation^65–67^. Moreover, they deliver a broad spectrum of reflectivity (Z) and precipitation (R) relationships (Z-R) for different radar frequencies, to broaden our understanding of water cycle processes and precipitation microphysics over the global oceans and to evaluate re-analysis and general circulation model data.

### OceanRAIN precipitation instrumentation

The backbone of the OceanRAIN project is the ODM470 optical disdrometer^30,59,61,68,69^. An infrared light emitting diode at 880 nm homogeneously illuminates the measuring volume of 120 mm length and 22 mm diameter. Hydrometeors passing this volume cause light extinction proportional to their cross sectional area. The detected light reduction is stored as an activation voltage^70^. During the integration time of 60 s all hydrometeors are counted and sorted into size bins ranging from 0.04 to 22 mm to obtain the PSD.

The OceanRAIN instrumentation is complemented by a cup anemometer to measure relative wind speed and a precipitation detector (IRSS88). The smallest particles detectable by the IRSS88 are of 0.39 mm diameter and therefore the same as the ODM470. The IRSS88 activates the disdrometer when precipitation occurs. This helps to increase the lifetime of the disdrometer optics and strongly reduces the occasional risk of artificial signals caused by vibration of the instrument due to sea state, ice-breaking activity, gusty winds or the ship’s engine. Further artificial signals due to cleaning of the optics and erroneous signals caused by birds or insects are also avoided by this technique. The OceanRAIN instruments are installed as high up as possible, preferably on the ship's mast and in front of the ship's funnel, to minimize the exposure to sea spray, wave water and soot. OceanRAIN records temporally discontinuous precipitation data but also includes the true-zero precipitation information. The no-precipitation signal carries important information for the analysis of precipitation frequency of occurrence, false alarm statistics as well as retrieval behavior analysis in satellite-derived precipitation products. A detailed description of the instrumentation and its measurement principles is provided in Klepp^30^.

Disdrometer calibration is performed before and after shipboard operation whenever the ships are accessible during port or maintenance periods. The twofold procedure first comprises of a lab hardware calibration of the optical axis and the adjustment of the reference voltage using steel ball bearings of increasing size. This is followed by an outdoor test site calibration using real rainfall events in wind speed conditions below 5 m s^−1^ and a reference rain gauge (ANS410) for accumulation comparison^71^. Both instruments typically differ in the order of 2% rain accumulation^30^. The calibration drift after shipboard operation is negligible in most cases because the reference voltage is continuously checked and adjusted during the cruises if necessary. Underway lens cleaning is required in two-month intervals on average as indicated by a reference voltage drop towards a quality check threshold of 3 Volt.

The ODM470 is specifically designed and further developed for shipboard operation under all-weather conditions. The main advantages of the ODM470 system over other existing disdrometers can be specified in five points, namely (1) the measurement volume of the disdrometer has a cylindrical shape causing precipitation particles to be independent of their incidence angle, (2) the measurement volume is always kept perpendicular to the local wind direction. This is achieved by a wind vane that pivots the instrument around a vertical axis. Consequently, local up- and downdrafts as well as turbulence induced by the ships superstructure cause a minimal impact on the measurement of the particle size distribution, (3) the high-resolution optical unit allows discriminating hydrometeors into 128 size bins with an logarithmically increased resolution towards smaller size bins, (4) an automated precipitation phase detection algorithm allows to process particle size distributions using either the rainfall or snowfall algorithm, and (5) the instrument is fully automated, robust and requires minimal maintenance during operation. Thus, it is ideally suited for long-term monitoring of precipitation over the global oceans in all-weather conditions.

The instruments successfully performed during snowfall at −30 °C in the Southern Ocean close to Antarctica, during torrential rainfall in the inner tropics with up to 367 mm h^−1^ rainfall and during severe sea states in mid-latitude storms.

### Data acquisition

**Figure 1:**
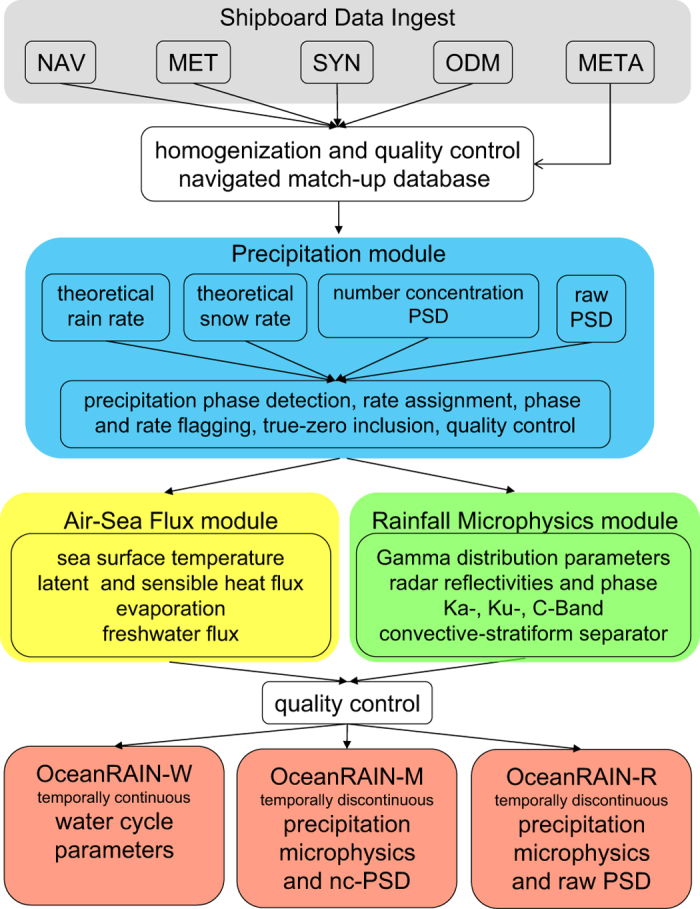
OceanRAIN data ingest and post-processing flow chart.

The data acquisition and data processing chain to derive water cycle and precipitation microphysical parameters is visualized in the flow chart of Fig. 1. Four universal time coordinate (UTC) time-synchronized data streams, using a joint IP time server, are recorded separately onboard the ships and are ingested into the OceanRAIN database. These are the temporally continuous shipboard navigational data (NAV), the automated underway surface atmospheric and oceanographic data (MET), the three-hourly manual synoptic weather observations (SYN) as well as the temporally discontinuous OceanRAIN ODM470 precipitation raw data (ODM) and metadata protocols on special weather and instrument conditions (META).

The NAV and MET files for all five German RVs (Table 1) are freely available from the DSHIP data center (http://dship.bsh.de/) of the Federal Maritime and Hydrographic Agency. For the Australian RV Investigator they are provided by CSIRO (Commonwealth Scientific and Industrial Research Organisation) and the Marine National Facility (MNF). The data from the American RV Roger Revelle is made available by University of Washington in Seattle. The luxury cruise liner MS The World supplies a limited set of meteorological data directly through ship contacts. Most of the ships measure air and dew-point temperature, bulk water temperature, relative humidity, air pressure, relative and true wind speed and direction, global radiation, visibility, ceiling, salinity and rain gauge data. The World Meteorological Organization (WMO) present weather (ww) and past weather (W1 and W2) codes including precipitation type and intensity observations (SYN) are additionally provided during daylight operation through the manned weather observatories onboard RV Polarstern and RV Meteor maintained by the German Weather Service. The synoptic code list is provided in Petty^26^.

The ODM files contain the temporally discontinuous PSDs in two raw data levels. During the integration time of 1 min, each particle occurrence is logged together with its properties into a binary file. From this 1-min resolution, data entries are automatically produced that contain the number of particles per bin size. This ODM raw data is stored into daily files that are designedly empty if no precipitation occurred. To further ensure the validity of true-zero minutes during post-processing, hourly system messages are written into log files to ensure the error-free operation of the system.

META on special weather and sea state is essential when monitoring precipitation to identify the reasons for instrument problems, outages or unusual spikes in the data. Therefore, within OceanRAIN, contacts were established with ship personnel or onboard weather observatories to log special weather and instrument occurrences. Problems during the ODM470 operation are reported via email. Consequently, data outages can be minimized by effective troubleshooting. This timely communication effectively speeds up the shipping of spare parts if required. The ODM data is transferred in delay-mode during port times.

**Figure 2:**
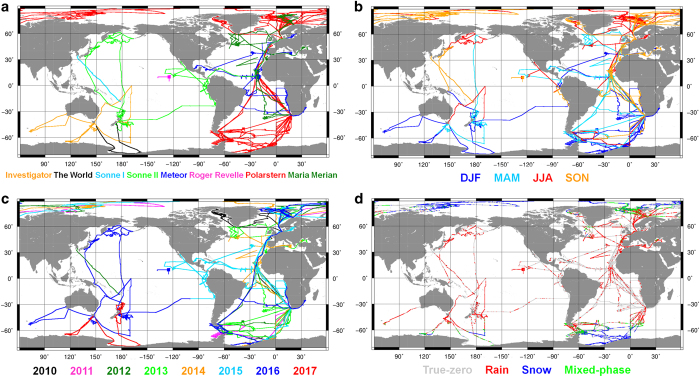
OceanRAIN data distribution for 8 ships. The panels show the data separated for (**a**), seasons (**b**), years (**c**) and precipitation occurrence for type (rain, snow mixed) and true-zeros (**d**).

Figure 2 provides an overview of the data recorded and processed for OceanRAIN version 1.0. The geographical distribution of all eight RVs (a) is color-coded for seasons (b), years (c) and precipitation phase occurrence including true-zero precipitation information (d). The dataset comprises a total of more than 6.83 million minutes of data with 696,740 min containing rainfall, snowfall or mixed-phase precipitation.

### Computational post-processing

The NAV, MET and SYN data files are delivered in a variety of non-uniform formats, file types and differ in the number and units of the measured and observed parameters. Thus, the first step in the OceanRAIN post-processing chain is to homogenize each incoming data record into a common interim format including uniform missing values. Values in knots or feet units are converted into metric units. Additionally, float and integer values are unified. From the NAV data files the latitude data is homogenized to degree North ranging from −90° to +90° and longitude in degree East ranging from −180° to +180°. Additionally, the ship’s heading is stored. Thereafter, all four data streams are collocated into a navigated 1-min resolution match-up database using the UTC time coordinate. Additional time coordinates are calculated to obtain local date and time, minute of the day, the Unix epoch time stamp in seconds since 01 Jan 1970, 00 UTC and the Julian date since 01 Jan 1994, 00 UTC.

During port times, parameters should be handled with care because the logging systems may be under maintenance, switched off or data might be unreliable due to heavy maintenance activity on the ship’s superstructure. Therefore, port times receive flag value 5 in the dataset (Table 2).

### Quality control

Cascading automated and visual inspection quality controls of the navigated match-up database throughout the OceanRAIN post-processing chain are of major importance because the data ingested is direct instrument output with no initial quality screening.

The NAV data is inspected for the existence of date and time recorded in UTC, latitude, longitude and ship heading. Non-sequential, missing or corrupted data is corrected where possible or flagged missing otherwise. Duplicate time steps are removed. The MET data is automatically inspected for out-of-range values following a protocol described in Klepp^30^. Erroneous data blocks are rigorously excluded from the data records using missing values while obvious errors (e.g. 0.00 hPa for air pressure) are replaced by interpolated estimates using previous and next minute data where possible. This is possible for all spatio-temporally homogeneous parameters (e.g. water temperature or navigation data) but not for highly variable and intermittent parameters (e.g. precipitation). Suspicious data is flagged and accepted or rejected using a visual screening procedure. This ensures that derived parameters (e.g. evaporation) that rely on a number of input parameters are only calculated once the input parameters successfully passed the quality control test. The SYN data is flagged for precipitation occurrences and visually compared to match the ODM precipitation occurrence. The ODM data is automatically inspected and visually corrected for unrealistically high single-minute spikes. Such occurrences are however not entirely deleted from the database. For traceability, they can be tracked by their non-zero number of particles and bins and their theoretical rain and snowfall rate. In the final precipitation rate assignment they obtain a true-zero value. The automatically derived precipitation phase probability is further inspected to agree with the derived theoretical rainfall and snowfall rate (see Precipitation module) to avoid unrealistic minute-to-minute variations in the precipitation record when mixed-phase precipitation is likely to be present. Details on these production steps are provided in the precipitation module section below. Again, any changes made can be tracked by the theoretical rainfall and snowfall rate and the precipitation phase flag. At this step, the META log protocols are thoroughly inspected and suspicious data is rigorously flagged or discarded. Such events include icing, riming, extreme sea states with wave water influence up to the mast level, true extreme precipitation occurrences, ice-breaking activity to monitor heavy instrument vibration as well as flocks of birds following the ship and instrument cleaning periods. Periods with ODM instrument malfunctions and erroneous data periods (e.g. icing or riming) are marked with value 4 in the flag1 parameter (Table 2). An open issue remains with the influence of the ship’s exhaust plume on air temperature measurements. Such temperature variations can be seen in the data and their occurrence can last from minutes to much longer time periods. A homogenized correction procedure should be ideally provided by the data operators for NAV and MET.

As a next step, the quality-controlled navigated match-up database is applied to the precipitation, air-sea flux and rainfall microphysics modules (Fig. 1) to derive precipitation occurrence and intensity for rain, snow and mixed-phase and the following parameters: turbulent heat fluxes, evaporation, freshwater flux and the PSD gamma distribution parameters, radar reflectivity parameters and convective-stratiform separators for rainfall.

### Precipitation module

Each minute with precipitation occurrence logged by the ODM contains the reference voltage, the relative wind speed measured by the cup anemometer at the disdrometer and the total number of particles and bins allocated. The data logger automatically produces two particle count PSDs, one for assumed rainfall and one for assumed snowfall, that contain the number of particles in each of the 128 size bins N(bin). The difference between the rain and snow PSDs results from different assumptions on the residence time allowance for liquid and frozen particles. The residence time is additionally logged for rain and snow but not used to derive parameters^30^. The 128 size bins logarithmically increase in resolution towards smaller particles. The first 12 bins ranging from 0.04 to 0.36 mm in size are not recorded because they are prone to contain artificial signals caused by ship vibration. From N(bin) the PSD density n(bin) in m^−3^ is calculated by dividing N(bin) by the product of the measurement volume (120 mm length, 22 mm diameter), the integration time (60 s) and the geometrical sum of the relative wind speed and the parameterized terminal fall velocity^68^. Normalization of n(bin) by the non-constant bin width leads to the number concentration PSD (m^−3^ mm^−1^). The 1-min resolution number concentration PSD (nc-PSD) is stored in the OceanRAIN-M files while the raw particle count PSD N(bin) is stored in the OceanRAIN-R files (raw PSD). Both files are temporally discontinuous and contain precipitation minutes only.

The calculation of the rainfall and snowfall rate P in mm h^−1^ requires the mass (liquid water equivalent) of the particles M(bin) and the terminal fall velocity V(bin) and is calculated after Pruppacher and Klett^72^ and Großklaus *et al*.^59^:

$$P=3600\sum_{bin=1}^{128}n\left(\mathrm{bin}\right)\cdot V\left(bin\right)\cdot M(bin)$$

For rainfall with near-spherical drop shapes and constant drop density, the parameterization of V(bin) and M(bin) is chosen after Atlas and Ulbrich^73^. For the complex problem of snowfall Lempio^70^ developed a common lump graupel parameterization based on the work of Hogan^74^, Brandes *et al*.^75^ and Macke *et al*.^76^, relating the measured cross sectional area to the maximum dimension of the particle. Therein, the product of *V*(bin) and *M*(bin) as a function of the cross sectional area remains in the same order of magnitude for a variety of different snow crystals. Because lump graupel is nearly spherical in shape it circumvents the need for a transfer function between the cross sectional area and the maximum dimension of the particles. This parameterization is limited to a size range of frozen hydrometeors from 0.39 to 9 mm diameter. This agrees with the OceanRAIN data: Klepp^30^ showed that larger snowflake events are rare. In addition, Klepp *et al*.^61^ reported that lump graupel was the most common type of precipitation over the cold-season Norwegian Sea.

Edge effects due to partially scanned particles are avoided by only registering particles with their center being within the volume. Coincidence effects of overlapping particles are statistically corrected^59,70^. The impact of artificial small droplets due to splashing on the disdrometer housing, snow blockage or wet lenses are minimized by the design of the disdrometer device. Additionally, a filtering is implemented that excludes signals exceeding a residence time threshold. A detailed discussion of the ODM data collection and algorithm chain including the parameterizations used for rainfall and snowfall are provided in the OceanRAIN technical reference^30^.

Each precipitation minute is ingested into the rainfall and snowfall algorithms to derive the theoretical rainfall and theoretical snowfall intensity in mm h^−1^. The final assignment for each minute of precipitation as rain, snow or mixed-phase precipitation including a precipitation phase probability is calculated using an automated OceanRAIN precipitation phase distinction algorithm^77^ and a follow-up quality control. This algorithm uses a logistic regression model after Koistinen and Saltikoff^78^ with the two recommended predictors air temperature and relative humidity and, additionally, the 99th percentile of the PSD particle diameter. The predicted precipitation phase has been compared against a manually determined precipitation phase from 3-hourly SYN of more than four years of OceanRAIN data from RV Polarstern covering all climatic regions and seasons over the Atlantic Ocean. In addition, all three rain, snow and mixed-phase probabilities are simultaneously determined from two individual rain and snow probability distributions independent from each other. Compared to the hitherto time-consuming manual precipitation phase distinction method, an accuracy of 91% is reached for rain and snow, and of 81.2% when including the mixed-phase precipitation phase. This accuracy reduction can be explained by the highly varying rain–snow fraction on the timescale of minutes within the phase transition zone from−3 °C to +6 °C. However, this is a major improvement over the manual precipitation phase distinction method because the SYN data is only updated in 3-hourly intervals during daylight. This new statistical method considerably speeds up the OceanRAIN data post-processing and, additionally, introduces an objective precipitation phase probability for rain, snow and mixed-phase precipitation at 1-min resolution^77^. This considerably reduces the earlier number of highly uncertain precipitation phase cases requiring visual inspection of atmospheric variables. Moreover, the OceanRAIN precipitation phase probability allows error characterizing precipitation satellite data sets to unveil systematic errors with respect to the precipitation phase.

Because no mixed-phase algorithm with precise rain-snow fraction exists yet, the snowfall intensity is assigned to mixed-phase minutes. Thus, the mixed-phase precipitation intensity is increasingly underestimated with increasing rain (liquid) fraction and inhibits the largest errors compared to the rainfall and snowfall values so that these precipitation intensities should be treated with care. Additionally, the mixed-phase to rain or snow transition may take place over a few meters or hundreds of meters in altitude. Because GPM and CloudSat retrievals discriminate between rain and snow, we decided to introduce mixed-phase precipitation to OceanRAIN so that the dataset can be stratified accordingly. The theoretical rain and snow intensities are kept in the database. This is of importance mainly for the mixed-phase precipitation because the difference between the theoretical rain and snow intensity provides information on the intensity uncertainty and therefore the liquid fraction during that minute. A mixed-phase precipitation algorithm is envisaged for future dataset releases.

As a next step, each minute in the data record is assigned two precipitation flags, called flag1 and flag2. Flag1 assigns the ODM precipitation phase or instruments condition (Table 2). Value 0 assigns a rainfall minute, 1 is snowfall and 2 is mixed-phase precipitation. Because the data in the OceanRAIN-M and OceanRAIN-R files is temporally discontinuous (precipitation-only events) there can be no true-zero value. However, the OceanRAIN-W files are temporally continuous and therefore contain the true–zero (no precipitation) information that is assigned value 3 in flag1. This is especially important for very light precipitation because two sources for 0.00 mm h^−1^ precipitation rates exist. First, a precipitation minute can either be zero because no precipitation occurred (true-zero; flag1=3) or, second, precipitation particles occurred with zero accumulation (flag1=0, 1, 2). Value 4 assigns an ODM malfunction and 5 represents port times as already mentioned above. This flag is very useful for users aiming to easily stratify the dataset for precipitation phase (see Usage Note section).

Flag2 further stratifies the precipitation events and intensities (Table 3). If the number of assigned bins and number of particles is smaller than three, a single-minute event is considered being an electronic artefact due to vibration issues. Therefore the precipitation rate is set to zero and a true-zero flag is assigned. However, this occurrence can be traced in the number of bins, number of particles and the theoretical rain and snow rate. All flag1=3 values correspond to flag2=10 values (true-zero, no precipitation). The ODM data contains many occurrences of minutes with less than 20 particles and less than 5 bins occupied. The corresponding precipitation rates are mostly insignificant or zero. The reason for these minutes could be either very light precipitation or strong vibration of the instrument resulting in spurious signals. Therefore, these values are assigned flag2=11 and the decision is left to the user whether to consider these insignificant precipitation intensities as real precipitation. Flag2 values between 12 and 17 stratify the precipitation intensity beginning from 0.00 mm h^−1^ (flag2=12) onward. Value 13 is introduced because the ODM is capable of measuring very light precipitation between 0.01 and 0.09 mm h^−1^, which is beyond the usual detection threshold of gauges of 0.1 mm h^−1^. Value 14 contains light precipitation from 0.1 to 0.99 mm h^−1^ that may be attributed to drizzle-like precipitation regimes, while value 15 contains moderate rates from 1.00 to 9.99 mm h^−1^ and value 16 covers strong precipitation rates of 10.00 to 49.99 mm h^−1^. Extreme precipitation cases are contained in class 17 with values from 50 mm h^−1^ onward. Class 16 and 17 precipitation events are almost certainly convective. Additionally, a physical convective-stratiform precipitation separator is introduced for rainfall (see Rainfall microphysics module section) that supports this assumption. Another way for users to stratify the data is to use thresholds in the number of bins and number of particles allocated.

The output of the precipitation module comprises precipitation occurrence including true-zero minutes, intensity, accumulation, precipitation phase and probability for rain, snow and mixed-phase, 99th percentile of particle diameter, theoretical rain and snow intensities, flag1, flag2, number of particles and number of bins as well as raw PSDs and number concentration PSDs.

### Rainfall microphysics module

The rainfall microphysics module enables direct access for experts as well as non-expert users to relevant parameters such as type of rainfall (convective versus stratiform), the main characteristics of the drop size distribution (DSD), and the resulting radar reflectivity at important frequencies for radar rainfall studies. In order to do so, the following state-of-the art approximations and tools are used. Please note that we processed 1-min PSDs for rainfall only due to the higher complexity of deriving such quantities for snow or mixed-phase precipitation. Research is already underway by our team to develop robust modules for snow and mixed-phase precipitation in future releases of the OceanRAIN dataset.

As demonstrated by the pioneering studies of Testud *et al*.^79^ and Bringi *et al*.^80^, the DSD can be accurately approximated by a normalized gamma distribution:

$$N\left(D\right)=N_{0}^{*}\frac{\Gamma \left(4\right)}{3.67^{4}}\frac{{\left(3.67+µ\right)}^{4+µ}}{\Gamma \left(4+µ\right)}{\left(\frac{D}{D_{0}}\right)}^{µ}\times exp\left[-(3.67+µ)\frac{D}{D_{0}}\right]$$

Normalized gamma distributions of rainfall are controlled by three parameters: *N*_o_* (also called *N*_*w*_ in the literature), the intercept of the distribution; *D*_*0*_, the median volume diameter of the distribution; and *μ*, the shape parameter of the distribution. The quality-controlled DSDs are fitted using this normalized gamma formulation. The three resulting parameters are provided in the OceanRAIN files given that the PSD has at least 10 size bins filled with data. Once *N*_o_* and *D*_*0*_ are retrieved, we classify the PSD as convective or stratiform rain using the physically-based convective-stratiform classification proposed by Thurai *et al*.^81^.

Although the normalized distribution framework is now commonly used in rainfall studies, the DSD is still sometimes approximated using the earlier standard gamma distribution (e.g., Tokay and Short^82^). Therefore, for users wishing to use this formulation to relate to earlier studies of statistical rainfall properties we have also performed standard gamma fits using the same threshold of 10 size bins and provided the intercept parameter N_0_ of the standard gamma distribution in the OceanRAIN files.

Williams *et al*.^83^ highlighted that since the three parameters of the normalized gamma distribution were not statistically independent, this was causing issues in the TRMM and GPM satellite retrievals of rainfall rate. A new framework has therefore been proposed by these authors, based on the two first moments of the mass spectrum: the mass-weighted mean diameter *D*_*m*_, which is the first moment of the mass spectrum, and *σ*_*m*_, the standard deviation of the mass spectrum. These two parameters have been calculated directly from *D*_*0*_ and *μ* following Williams *et al*.^83^; they are included in the OceanRAIN files.

Finally, we have estimated radar reflectivity *Z*, differential reflectivity *Z*_*DR*_, and specific differential phase *K*_*DP*_ at different frequencies (Rayleigh at 3 GHz, C-band at 5.6 GHz, Ku-band at 13.6 GHz and Ka-band at 35 GHz) using the pyTmatrix tool developed by Leinonen^84^. These parameters help users interested in applications such as satellite radar retrievals of rainfall rate, GPM satellite radar validation, or high-resolution model evaluation. The main assumptions of such T-matrix calculations are the drop shape model and the standard deviation of the canting angle. In order to produce the radar parameters for the OceanRAIN dataset, the drop shape model from Thurai *et al*.^85^ and a standard deviation of canting angle of 20° were used, both settings are recommended in pyTmatrix.

### Air-Sea Flux module

The precipitation (P) parameter in OceanRAIN is complemented by the turbulent heat fluxes and the evaporation (E) to obtain the freshwater flux (E-P). The evaporation is derived from along-track ship data using a bulk formulation according to Fairall *et al*.^86^. This requires the shipboard air temperature, relative humidity, water temperature and absolute wind speed at minute-resolution that are measured at varying heights on different ships (Table 4). From these values the sea surface temperature (SST) and latent as well as sensible heat fluxes are parameterized using the Coupled Ocean-Atmosphere Response Experiment (COARE) bulk flux algorithm^86^ version 3.0, which is an updated version of Fairall *et al*.^87^ including higher latitudes and stronger wind conditions.

The true wind speed measurement *u* is reduced to the 10 m reference height *u*10 using a neutral-layer logarithmic wind profile after Tennekes^88^. The relative humidity serves to determine the specific air humidity *qa* (g kg^−1^) and the saturation specific humidity at the sea surface *qs* (g kg^−1^) following Murphy and Koop^89^.

The bulk water temperature T_water_ measured in sea water inlets between two and seven meters depth (Table 4) is used to calculate the sea surface temperature SST by applying the cool skin parameterization ΔT_water_ after Donlon *et al*.^90^:

$${\Delta \text{T}}_{\text{water}}=-0.14-0.3\exp\left(\frac{u10}{3.7}\right)$$

The warm-layer effect on the SST is not implemented in OceanRAIN as most ships lack providing a continuous diurnal cycle measurement of the surface radiation budget. Instead, OceanRAIN contains a warm-layer flag (WLF) that indicates the quality of the derived SST product. The warm-layer effect may influence the SST at wind speeds below 6 m s^−1^ and global radiation exceeding 50 W m^−2^ (WLF=2). At wind speeds below 2 m s^−1^ the SST is influenced by convective and molecular heat transports and a strong warm-layer (WLF=1) may be present^90,91^. In contrast, high wind speeds beyond 6 m s^−1^ destroy the warm-layer and the effect becomes insignificant (WLF=0).

The COARE algorithm iteratively estimates the stability-dependent scaling parameters for the drag transfer coefficient C_d_, the latent heat flux transfer coefficient C_e_ and the sensible heat flux transfer coefficient C_h_. Additionally using the potential temperature *θ* lead to the bulk flux calculation of the latent heat flux *H*_*l*_ and sensible heat flux *H*_*s*_ in W m^−2^ after Brunke *et al*.^92^:

$$H_{l}=\rho_{a}\;L_{e}\;{\text{C}}_{\text{e}}\;u_{10}(q_{s}-q_{a})$$

$$H_{s}=\rho_{a}\;C_{p}\;{\text{C}}_{\text{h}}\;\;u_{10}(SST-\theta )$$

where $\rho_{a}$ is the air density, *C*_*p*_ is the specific heat at constant pressure and *L*_*e*_ is the SST-dependent latent heat of vaporization. The additional sensible heat flux caused by the precipitation is implemented after Gosnell *et al*.^93^. However, the cooling rate due to snow or mixed-phase precipitation is not implemented in the Gosnell calculation but is expected to have additional influence.

The evaporation in mm h^−1^ is derived from the turbulent latent heat flux after Fairall et al.^87^ by:

$$E=H_{l}/(L_{v}\;\rho_{o})$$

where $\rho_{o}$ is the density of ocean freshwater as a function of temperature. The specific air humidity *q*_*a*_ is adjusted if it exceeds the value of *q*_*s*_ using *q*_*a*_=*q*_*s*_−0.01 (g kg^−1^) to avoid unrealistic negative turbulent latent heat fluxes below −25 W m^−2^ and in turn unrealistic negative evaporation values.

The difference between the evaporation E and the precipitation P yields the ocean surface freshwater flux E-P in mm h^−1^ into the atmosphere.

After a final quality and consistency check, all measured, observed and derived parameters are written into the temporally continuous 1-min resolution OceanRAIN-W data files (Data Citation 1). All precipitation relevant parameters are stored into the temporally discontinuous 1-min resolution OceanRAIN-M (Data Citation 2) and OceanRAIN-R (Data Citation 3) files that contain precipitation events only and additionally hold the number concentration PSD and the raw particle count PSD. The data is available for each of the eight ships in netCDF and ascii formats (see Data Records section). The OceanRAIN-1.0 dataset provides 696,740 minutes with 414,807 rain, 232,358 snow and 49,575 mixed-phase precipitation including 4,699,282 true-zero minutes (Fig. 1) for all seasons over the global oceans including the high-latitudes. The true-zero precipitation values are important for validating false alarm statistics in satellite products and to analyze the underlying retrieval characteristics. Therefore, OceanRAIN-1.0 tremendously increases the amount of available high quality data.

### Code availability

The OceanRAIN code is available upon request through a scientific cooperation agreement.

## Data Records

OceanRAIN provides users with three dataset versions (OceanRAIN-W, Data Citation 1; OceanRAIN-M, Data Citation 2; OceanRAIN-R, Data Citation 3) for each of the eight ships in the OceanRAIN fleet. The aim is that users can choose the dataset version that best meets their research needs. The along-track point data covers the global oceans from −90° to 90°N latitude and −180° to 180°E longitude from 10 June 2010 to 10 April 2017. The files are named following the convention OceanRAIN-id_RV-ship-name_ship-identifier_UHAM-ICDC_startdate-enddate_v1_0 with .nc or .ascii as file extension. The ship names, ship identifiers as well as start- and end dates are listed in Table 1. The files are produced at Universität Hamburg (file identifier UHAM) and are hosted at the Integrated Climate Data Center (ICDC).

The OceanRAIN-W contains the 1-minute resolution water cycle components of evaporation, precipitation and the freshwater flux along with all meteorological and oceanographic state variables required to derive these fluxes. The dataset is continuous in time and contains 73 parameters and more than 6.83 million minutes of data (Table 5 (available online only)). Typical applications for OceanRAIN-W comprise process studies and statistical analysis as well as satellite validation and re-analysis or model evaluation. OceanRAIN point data can serve as the surface reference and can be collocated with satellite or model data to analyse and improve their error characteristics. Therefore, it is important to highlight that the RVs sampled on the global oceans during all seasons including the cold-season Southern Oceans.

OceanRAIN-M and OceanRAIN-R focus on minutes containing precipitation and are therefore discontinuous in time. Both datasets comprise 37 precipitation-relevant parameters plus the 128 size bin number concentration PSDs (OceanRAIN-M) and raw number count PSDs (OceanRAIN-R) for 696,740 min in total with rain, snow or mixed-phase precipitation (Table 6 (available online only)). The precipitation-related parameters are identical in the three versions of the dataset. Applications for these datasets especially comprise satellite retrieval performance evaluation for liquid and solid precipitation. For this purpose, OceanRAIN-M and OceanRAIN-R supply the user with a convective versus stratiform precipitation classification and contain the main PSD characteristics and the radar reflectivities at important frequencies for radar rainfall studies. This is of special importance for users aiming at TRMM, CloudSat and GPM product and retrieval validation because these satellite missions carry spaceborne radars.

The OceanRAIN version 1.0 datasets are freely available as netCDF and ascii files from the Climate Computing Center (DKRZ) repositories of the World Data Center for Climate (WDCC) and are provided in the Data Citations section. They can also be retrieved through the Integrated Climate Data Center (ICDC) at the University of Hamburg via http://icdc.cen.uni-hamburg.de/1/daten/atmosphere/oceanrain/ and through the OceanRAIN website http://www.oceanrain.org/. A ReadMe file is provided at each of the data repositories to assist the user on ingesting the data.

## Technical Validation

### Precipitation occurrence and accumulation

The OceanRAIN-W absolute precipitation occurrence (including true-zero minutes) across all latitudes and ocean basins is 14.8%. Separated by precipitation phase, rainfall occurs in 8.8%, snowfall in 4.9% and mixed-phase precipitation in 1.1% of the time. A large fraction of this percentage comprises very light precipitation. Without extremely light precipitation minutes with intensities less than 0.01 mm h^−1^, that are set to zero in the database, the global occurrence reduces to 9.8% (rainfall 6.9%, snowfall 2.6% and mixed-phase precipitation 0.3%). Excluding intensities below 0.1 mm h^−1^, the equivalent of what a typical rain gauge would be able to measure, the global occurrence reduces to 6.2% (rainfall 4.9%, snowfall 1.2% and mixed phase precipitation 0.1%). This demonstrates that the oceanic precipitation occurrence is mainly driven by very light precipitation. However, the rainfall percentage as a function of precipitation threshold is fairly stable in the tropics and subtropics (e.g. tropics: 4.5% including true-zeros, 3.9% less than 0.01 mm h^−1^ rainfall occurrences and 3.1% for events larger than 0.1 mm h^−1^). In contrast, the mid- and high-latitudes are dominated by very light precipitation (e.g. northern high-latitudes: 16.0% including true-zeros, 8.5% less than 0.01 mm h^−1^ rainfall occurrences and 4.3% for events larger than 0.1 mm h^−1^).

The global relative precipitation occurrence (all precipitation minutes set to 100%) contains 59.5% rainfall (70.6% less than 0.01 mm h^−1^ and 79.9% exceeding 0.1 mm h ^−1^). Snowfall occurs in 33.4% (26.5 and 18.8%) of the time and mixed-phase precipitation in 7.1% (2.8 and 1.3%) of the time. The increasing relative rainfall percentages at rates less than 0.01 mm h^−1^ and those exceeding 0.1 mm h ^−1^ are caused by the strong decrease of the corresponding snowfall and mixed-phase occurrences.

As expected, the very light precipitation has little effect on the precipitation accumulation. In total the OceanRAIN 1.0 dataset contains 8867.6 mm precipitation accumulation. Rainfall dominates with 92.1% while snow contributed 7.4% to this accumulation and mixed-phase as little as 0.5%. These numbers change only marginally for accumulations from intensities above 0.1 mm h^−1^.

**Figure 3:**
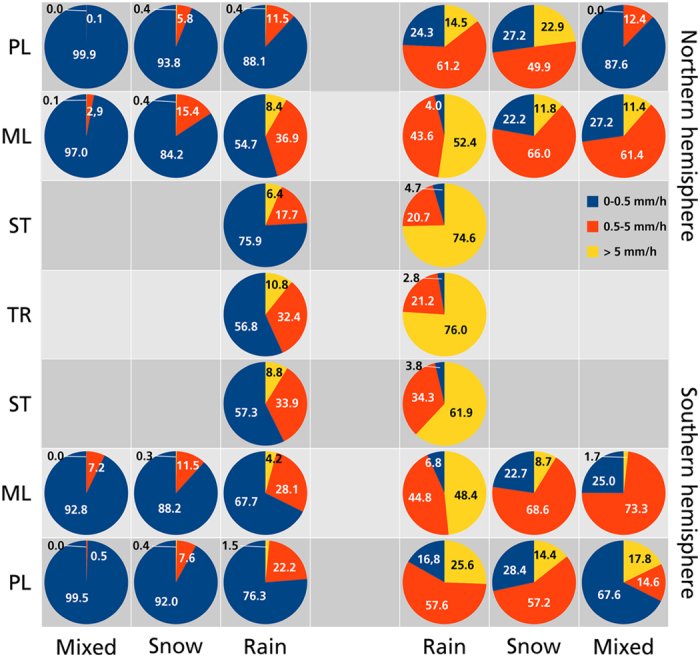
Latitudinal phase-dependent precipitation occurrence and accumulation. Relative percentage of precipitation occurrence (left) and contribution to accumulation in percent (right) for rain, snow and mixed-phase precipitation as a function of precipitation intensity (colors) and latitudes (top to bottom). For both hemispheres TR denotes tropics (±10°), ST subtropics (±10–35°), ML mid-latitudes (±35–60°) and PL polar-latitudes (±60–90°).

To get an overview of the sampled precipitation over the global oceans, Fig. 3 depicts the relative percentages and absolute accumulations for rain, snow and mixed-phase precipitation as a function of precipitation intensity through the latitudinal belts of both hemispheres. The figure does not account for seasonal variations, e.g. the meridional shift of the intertropical convergence zone. Again, light precipitation dominates the precipitation occurrence (chosen here from 0 to 0.5 mm h^−1^). Light precipitation occurs least often in the inner tropics with 56.8% of the time. The light rainfall fraction largely increases towards the poles and reaches 88.1% in the northern polar latitudes. The occurrence of light snow and light mixed-phase precipitation below 0.5 mm h^−1^ ranges between 84.2 and 99.9%. Precipitation between 0.5 and 5 mm h^−1^ mainly occurs as rainfall and varies between 11.5% in the northern polar latitudes and 36.9% in the northern mid-latitudes. Additionally, the polar and mid-latitudes of both hemispheres contribute between 5.8 and 15.4% of snowfall between 0.5 and 5 mm h^−1^. Rainfall beyond 5 mm h^−1^ occurs in 0.4% of the time in the northern polar latitudes and increases towards a maximum of 10.8% in the tropics. However, this occurrence of 10.8% contributes by 76.0% to the total rainfall accumulation in the tropics. These values remain high in the subtropics (61.9 and 74.6%) and the mid-latitudes where this rainfall type contributes between 48.4 and 52.4% to the accumulation. Expectedly, light precipitation has little effect on the accumulation and varies between 2.8 and 6.8%. Towards the polar-latitudes the light rainfall accumulation increases up to 24.3% and reaches 28.4% for snowfall and 87.6% for mixed-phase precipitation.

### Variability of water cycle components

**Figure 4:**
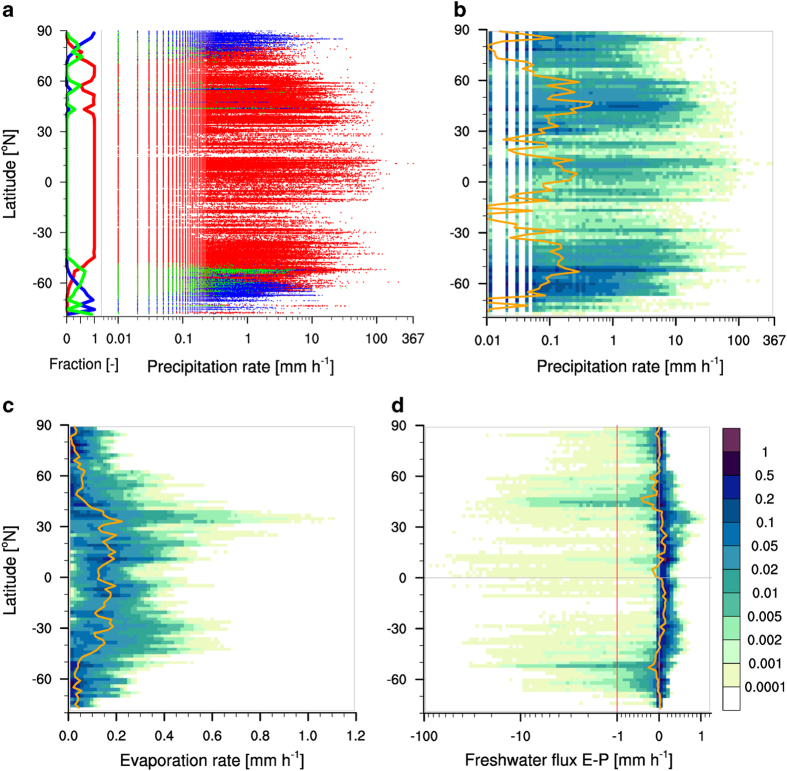
Variability of water cycle components. Panel (**a**) shows latitudinal fraction the precipitation phase with red for rain, blue for snow and green for mixed phase. The scatter diagram shows individual precipitation minutes for phases against latitude as a function of precipitation rate (mm h^−1^). Panels (b-d) show joint histograms of precipitation rate (**b**), evaporation rate (**c**) and the resulting freshwater flux (**d**). Units are mm h^−1^, orange line indicates bin-wise mean for 2° latitude bands. Frequency of occurrence in % of all cases is shown with colors. Note the logarithmic axis scaling in panel d for E-P less than −1 mm h^−1^.

An overview on the variability of the water-cycle components in the OceanRAIN-W database across the latitudes is depicted in Fig. 4. The latitudinal precipitation fraction in Fig. 4a shows the occurrence of mixed-phase precipitation poleward of 40°N and 45°S. Snowfall occurs poleward of 44 °N and 50 °S. Solid precipitation is the predominant phase beyond 80 °N and 53 °S. The disparity of the latitudes on both hemispheres is due to the boreal summer sampling of the RVs in the Arctic while the RVs sampled all seasons in the Southern Oceans. The latitudinal precipitation phase as a function of precipitation intensity comprises all 414,807 minutes of rainfall, 232,358 snowfall minutes and 49,575 min with mixed-phase precipitation. The highest rainfall intensities occur in the tropics between −10° and +15° with a record value of 367 mm h^−1^ onboard RV Meteor on 14 September 2015 in the tropical Atlantic. During 68 minutes of rainfall, the tropical cluster accumulated about 40 mm of rainfall, which mainly occurred during nine consecutive minutes with rainfall exceeding 50 mm h^−1^.

The joint histogram of the precipitation intensity in Fig. 4b shows that subtropics are characterized by significantly fewer rainfall events while individual events reach intensities of up to 100 mm h^−1^. The mid-latitude rainfall begins at approx. 35° latitude on both hemispheres. The rainfall intensities in the mid-latitudes are comparable with those measured in the tropics. First, this indicates the importance of mid-latitude cyclones that contribute a major fraction of the global rainfall with high intensities at high wind speeds. Second, this may indicate an undersampling of the intertropical convergence zone rainfall in the OceanRAIN dataset. Ship personnel metadata often reports thunderstorm and torrential rain in the vicinity of the ships while the ship track remains largely dry. Comprehensive sampling of the inner tropical convection cells requires more ships and more years of data collection. The next version of the OceanRAIN dataset will largely improve this sampling. This is also supported by the bin-wise mean for 2° latitude bands. The zonal mean curve shows a strong latitudinal fluctuation and therefore is strongly precipitation event driven. The climatology is only met where the sampling is high, as indicated by the darker colors of the frequency of occurrence in percent. In turn, the zonal mean does not meet the climatology value wherever the sampling is low (e.g. in the inner tropics). The transition from rainfall to snowfall and mixed-phase precipitation occurs at 50°S and 44°N, respectively. However, most of the northern hemisphere cold-season precipitation is measured at latitudes northward of 70°N. This is because the Southern Oceans are sampled in OceanRAIN during all seasons with three ships including the austral winter cruise of RV Polarstern during August 2013. This cruise alone contributed about 40,000 minutes with snowfall at temperatures as low as −30 °C. In contrast, the northern hemisphere is sampled more infrequently by two ships and during the boreal summer only. However, it has to be noted that RV Polarstern crossed the North Pole repeatedly in September. Although there is strong research focus on the Southern Oceans, it is envisaged to improve the northern high-latitude sampling including the cold-season.

Between 0° and 10° north of the equator the evaporation shows a prominent local minimum where the highest rainfall rates occur (Fig. 4c). The evaporation is strongest in the subtropical belts between approx. 20° and 45° on both hemispheres and reaches values of about 1.2 mm h^−1^. Toward the poles the evaporation rapidly decreases to values below 0.1 mm h^−1^. The dense sampling of the evaporation leads to a zonal mean curve that approaches the climatology with a local minimum of 0.13 mm h^−1^ in the inner tropics followed by values in the order of 0.2 mm h^−1^ in the subtropics. The mid-latitudes show values around 0.05 mm h^−1^ with a decreasing trend towards the poles.

For the freshwater flux (precipitation minus evaporation), positive values denote a net flux of moisture (evaporation) into the atmosphere, while negative values indicate a net flux into the ocean (precipitation). The high temporal resolution of the data results in a long tail of the distribution towards negative values whenever intense precipitation occurred (Fig. 4d). The negative freshwater flux regimes are located in the inner tropics (−0.15 mm h^−1^) and the mid-latitudes (−0.2 to −0.4 mm h^−1^). They are strongly driven by events with intense precipitation. The maxima occur in the subtropics of both hemispheres with values of about 0.1 mm h^−1^. Again, they meet the climatology because of the dense sampling. Towards the poles the freshwater flux strongly decreases to mean values around zero.

### Precipitation microphysics

**Figure 5:**
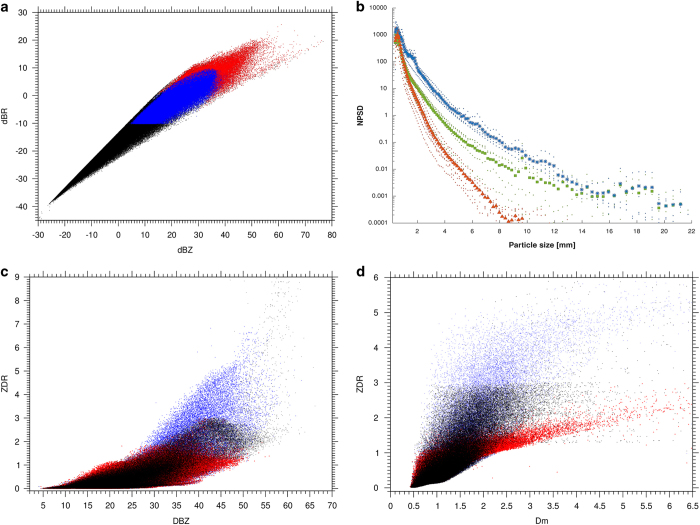
Precipitation microphysics in the OceanRAIN database. Panel (**a**) shows the Rayleigh reflectivity log(Z) and log (R) distribution of rainfall for all 696,740 events (black), 139,557 stratiform events (blue) and 15,823 convective events (red), (**b**) the mean number concentration PSDs (thick marks) and their latitudinal variability (thin marks) for all rainfall (red), snowfall (blue) and mixed-phase precipitation (green), (**c**) the DBZ versus ZDR relationship for rainfall only (C-Band black, Ka-Band red and Ku-Band blue), and (**d**) the same as in (**c**) using Dm versus ZDR.

The precipitation microphysics of the OceanRAIN-M database are evaluated in Fig. 5. The log-normal distribution of precipitation exhibits a tremendously high number of small precipitation particles and thus explains that light precipitation occurs much more frequently compared to high precipitation intensities. While the light precipitation intensities contribute most to the precipitation occurrence, moderate to heavy precipitation intensities contribute most to accumulation. This highlights the need to resolve the PSD at both extreme ends which is why a logarithmic size binning is introduced to the ODM470. To demonstrate this, Fig. 5a shows the log(Z) and log(R) values in dBZ and dBR for all 696,740 rainfall events, as well as for subsets of stratiform (139,557 minutes) and convective rainfall (15,823). Fig. 5a resembles Fig. 1 in Dölling *et al*.^94^ that consists of 200,548 1-min spectra of cold air advections in Hamburg, Germany. The black part of the distributions holds the number of 1-min spectra with less than 10 bin-sizes occupied in the PSD. The blue part contains the stratiform-classified minutes, with rainfall rates ranging from 0.1 to about 10 mm h^−1^. The red points show the convective-type rainfall. Convective rain rates are found between 0.5 to 367 mm h^−1^ in our dataset. The distribution reaches from one-drop spectra at 0.39 mm (−40 dBR, −35 dBZ) to extreme precipitation events at 20 dBR and 55 dBZ. The tail of the distribution reaching 20 dBR and 80 dBZ contains the one-drop spectra at 10 mm diameter (Fig. 5b). From all enclosed points or any subset of the dataset it is possible to derive Z (Rayleigh reflectivity) – R (rainfall rate) relationships to constrain, validate or improve radar-derived and modelled rainfall rate estimates.

Figure 5b depicts the mean number concentration PSDs (bold marks) and their latitudinal variability (small marks) as used in Fig. 3 for all 696,740 precipitation 1-min spectra (414,807 minutes of rainfall, 232,358 snowfall and 49,575 mixed-phase precipitation). The number concentration PSDs resemble those in Klepp^30^. In contrast to this early version, the OceanRAIN 1.0 dataset comprises a 12-fold increase in the number of 1-min rainfall spectra, a 6-fold increase for snowfall and 2.5-fold for mixed-phase precipitation. The rainfall PSD peaks at a diameter of 0.39 mm with a number concentration of about 1000 m^−3^ mm^−1^ and steeply decreases down to about 0.003 m^−3^ mm^−1^ for the largest drops of about 6.5 mm diameter. Single drops, however, can reach a diameter of about 9.7 mm and were measured during convective rainfall in the tropics. The snowfall curve peaks at 1100 m^−3^ mm^−1^ and descends more gradually towards largest measured snowflakes of 22 mm in diameter. The mixed-phase precipitation curve resembles that of rainfall for diameters between 0.39 and 1 mm diameter. For diameters up to 6 mm it lies between the curves for rainfall and snow. For larger diameters the curve converges towards that of snowfall and reaches the snowfall curve at 14 mm diameter. The largest snowflakes in the mixed-phase distribution reach 19.5 mm in diameter. The number concentration at 0.39 mm is higher for frozen particles than for rain drops because rain drops tend to evaporate faster than frozen particles.

Figure 5c,d summarize the differential reflectivity (Z_dr_) versus reflectivity (Z) and Z_dr_ versus mass-weighted mean diameter (Dm) relationships for simulated C-Band, Ku-Band and Ka-Band radar frequencies. They resemble the theoretical curves shown in Fig. 7.20 and Fig. 7.23 in Bringi and Chandrasekar^95^. The ODM470 Z_dr_ is the simulated difference in returned energy between the horizontally and vertically polarized pulses of a radar under the assumption of different radar frequencies. Hence, Z_dr_ is a measure for the oblateness of the rain drops. As expected, increasingly bigger rain drops in Fig. 5d are associated with increasing Z_dr_, in other words more oblate rain drops. These figures also highlight the large variability of co-variation of polarimetric radar variables with radar frequency.

### Precipitation validation using radar, gauge and ODM470 data

**Figure 6:**
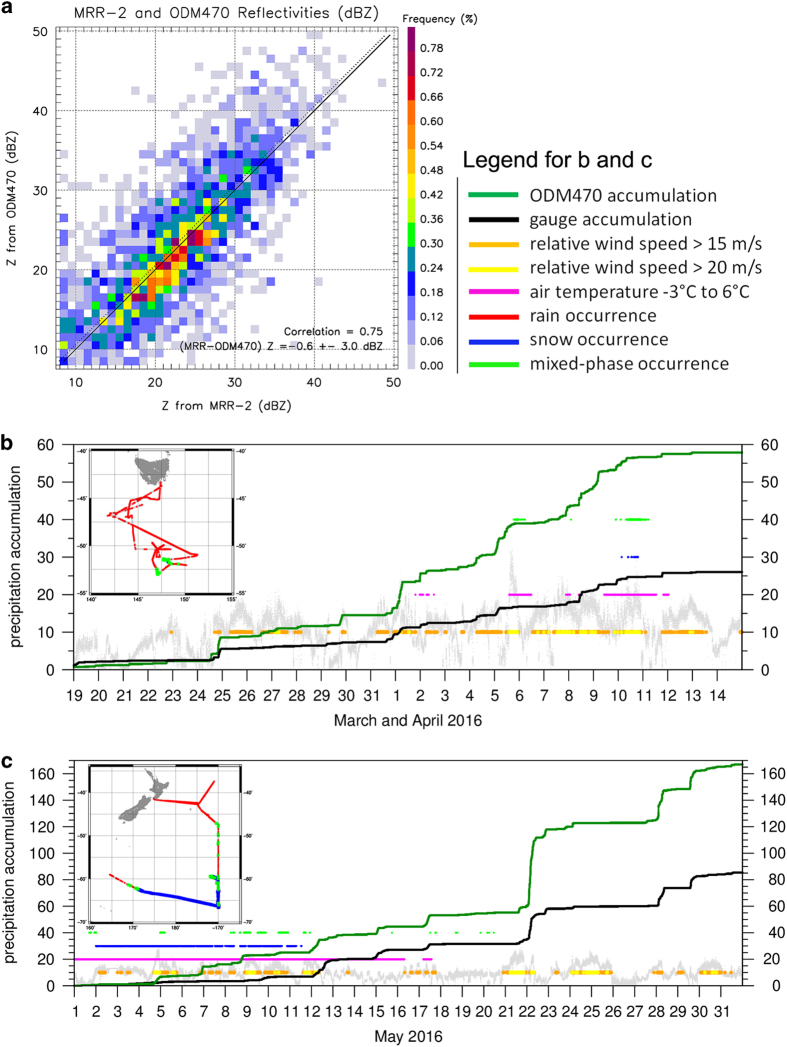
Precipitation validation using radar, gauge and ODM470 data over the Southern Ocean. Panel (**a**) shows the joint frequency distribution (in %) of measured MRR-2 reflectivities and reflectivities simulated using the ODM470 PSDs. The solid line is the 1:1 line, and the bias is shown as a dashed line. (**b**) The corresponding precipitation accumulation for the ODM and gauge along with relative wind speed for the time period from 19 March to 14 April 2016. (**c**) same as (**b**) but for May 2016. The inserted maps show the ship track positions and precipitation occurrence for rain, snow and mixed-phase together with true-zeros (grey). The legend refers to Figures b) and c).

In the introduction to this paper we made the claim that the OceanRAIN ODM470 disdrometer was able to mitigate the significant issues commonly encountered using rain gauges or off-the-shelf disdrometers not specifically designed for shipboard operation. During the CAPRICORN experiment from RV Investigator, a micro-rain radar^96^ (MRR-2) was deployed next to the long-term installed ODM470 and a conventional RM Young Rain Gauge^97^ type 50202 from 19 March to 14 April 2016 measuring precipitation over the Southern Ocean in the southeastern Australian section. The MRR-2 measures vertical profiles of radar reflectivity at 24 GHz. The improved Doppler spectral processing technique from Maahn and Kollias^98^ has been used to estimate reflectivity. This setup was therefore the perfect opportunity to substantiate these claims. Fig. 6a shows a joint frequency distribution of reflectivities simulated from the ODM470 PSDs using the pyTmatrix tool and those directly measured by the MRR-2 radar. There is no expectation for such comparisons to be perfect: the first useful radar range bin was about 100 m above the ODM470 disdrometer, the sampling volume of a disdrometer and a radar are very different (although this effect is partly mitigated using 1-minute averages), and the T-matrix simulations of radar reflectivity from disdrometer measurements are not perfect due to assumptions made about the drop shape model and standard deviation of canting angle (errors of 2 dB or more can be expected). Although minimized to the extent possible, through the installation in the mast and the instrument design, nevertheless typical artefacts associated with high winds, drop breakup on the instrument, flow distortion from the ship superstructure, should all produce visible artefacts on such comparisons^30^. As can be seen from Fig. 6a, the agreement is very good, without any noticeable systematic issue at different reflectivity levels. The correlation between the two measurements is 0.75, which is excellent when considering the previously discussed large differences in sampling volumes. The mean bias in reflectivity is −0.6 dB (ODM470 reflectivity simulations are on average 0.6 dB higher than MRR-2 reflectivities), which is smaller than the expected errors inherent to T-matrix assumptions, and probably smaller than the expected error on the MRR-2 calibration itself. The standard deviation of the difference between the two reflectivities is 3 dB, which again is small when considering all sources of uncertainties affecting these comparisons. Overall, these comparisons clearly showcase the high quality PSD measurements collected by the ODM470 instruments in a wide range of radar reflectivities.

As a second step of the intercomparison, Fig. 6b shows the total accumulation in mm from 1-min precipitation rates of the ODM470 compared to the conventional ship rain gauge over the same time period. Because wind speed belongs to the most influential factors to explain significant undercatch of gauges during rainfall events, the relative wind speed in m s^−1^ is also depicted. Additionally, 4 bars are added indicating when the relative wind speed was above 15 m s^−1^, above 20 m s^−1^, the air temperature was between −3° and +6°C, snowfall occurred or mixed-phase precipitation was present. The cruise track in the Southern Ocean south of Tasmania is depicted in the geographical map inset in Fig. 6b. Rain occurred during 9481 minutes while solid precipitation was relatively rare (773 minutes with mixed-phase and 38 with snow). True-zero minutes are shown in grey. The overall precipitation occurrence is high with 27%. The relative wind speed was for most of the time between 10 m s^−1^ and 20 m s^−1^. The accumulation of the ODM470 reached 57.2 mm while the gauge measured 24.9 mm, resulting in a significant gauge undercatch of 56.5%. However, the accumulation curves are in phase for most time steps because of the few solid precipitation occurrences.

To demonstrate that such high undercatch values are not specific for this time period, the third step of the intercomparison in Fig. 6c shows the follow-up RV Investigator cruise out of Wellington from 1 to 31 May 2016 in the Southern Ocean south and southeast of New Zealand, as depicted in the geographical map inset. This cruise is distinguished by its exceptionally high precipitation occurrence of 40% including a large amount of solid precipitation. 6333 out of 44,640 min contained snowfall and 1045 mixed-phase precipitation while rain fell in 10,381 min. During periods of mixed-phase precipitation and snowfall the gauge is expected to detect few to no precipitation. Snowfall during low wind speed conditions will clog the orifice. Once temperatures rise above the freezing level, melting snow produces artificial, delayed meltwater signals. This effect occurs from 4 to 11 May 2016 in Fig. 6c when the accumulation curves for the ODM470 and the gauge are not in phase because of the snowfall and mixed-phase occurrence at wind speeds frequently exceeding 15 m s^−1^. During this period, the ODM470 accumulated 25.4 mm in total while the gauge measured 5.3 mm, equivalent to 79.3% of undercatch. However, the gauge clearly misses the events seen in the ODM470 accumulation and instead shows a slight, almost linearly increasing signal with time, except for an accumulation phase in the second half of 9 May during which the ODM470 did not record significant precipitation. Instead, during the evening of 8 May, the air temperature was rising from freezing temperatures to 1.4 °C and falls significantly below freezing level again on 10 May. Therefore, melting snow in the gauge orifice explains this gauge accumulation leading to an artificial precipitation event of 2.6 mm. Without this melting event, the undercatch of the gauge would reach 90%.

On 22 May, an intense rain event occurred from 00:40 UTC to 11:31 UTC with a maximum ODM470 precipitation rate of 39.4 mm h^−1^ resulting in an accumulation of 51.4 mm in the ODM470 data while the gauge accumulated 18.0 mm (54.3% undercatch) at about 12 °C. However, high relative wind speeds exceeding 15 m s^−1^ and even 20 m s^−1^ were present during the time period when the ODM470 recorded peak accumulation rates.

During the whole month of May 2016, the gauge missed 48.9% of the precipitation measured by the ODM470 (167.1 mm). The 7.6% better performance of the gauge during May 2016 of 48.9% compared to 56.5% undercatch during March to April 2016 results from the overall lower relative wind speed regime during May. Whereas during May 2016 53% of the rainfall, 73% of the snowfall and 86% of the mixed-phase precipitation fell at wind speeds exceeding 10 m s^−1^, higher relative wind speeds occurred from March to April 2016 (78, 100, and 98%). Adding to the solid precipitation undercatch, the gauge received significantly less precipitation during rainfall because 26% of the rainfall events occurred at winds exceeding 15 m s^−1^ in May while this was the case for 40% in March and April 2016.

This observed gauge-undercatch by approximately 50% for rainfall at high relative wind speeds is common in the OceanRAIN database. For snow, the gauge undercatch is in the order of 90% and often reaches 100%. The gauge reliability for rainfall improves for all ships with decreasing wind speeds and reaches good to perfect agreement if the wind decreases significantly below 5 m s^−1^. However, it has to be considered that in 83% of the time, the relative wind speed in the entire OceanRAIN database of eight ships exceeds the critical threshold of 5 m s^−1^ when precipitation is present. This value does not vary with precipitation phase (84% rainfall, 80% snowfall, 83% mixed-phase precipitation). Thresholds exceeding 15 m s^−1^ still reach 27% for rainfall, 17% for snow and 24% for mixed-phase precipitation.

### Usage Notes

Users requiring surface reference data of precipitation including PSDs should use the OceanRAIN-M (number concentration PSD) or OceanRAIN-R (particle count PSD) files both of which are discontinuous in time. The continuous in time OceanRAIN-W data should be used if the true-zero precipitation information is needed or if additional meteorological and oceanographic parameters are required such as the along-track turbulent heat fluxes, the evaporation or the freshwater flux.

### Usage of precipitation flags

Two precipitation flags are consistently used in all three versions of the dataset in order to discriminate the precipitation phase and intensity. Flag1 (Table 2) assigns a value for rainfall (0), snowfall (1), mixed-phase precipitation (2) or no precipitation (true zero) (3) to each minute. Note that the data records in the OceanRAIN-M and OceanRAIN-R files are discontinuous in time and thus contain precipitation minutes only. However, it is important to note, that very light precipitation may result in a precipitation rate of 0.00 mm h^−1^ (flag1=0, 1, 2) due to an insignificant number of particles measured. In contrast to these events, during true-zero precipitation occurrences in the OceanRAIN-W files (flag1=3), the ODM470 relative wind speed and reference voltage is set to −888.88 in order to distinguish these minutes from the missing value (−999.99). Flag1 also indicates instrument outages with a value of 4 and harbour times with a value of 5.

Precipitation flag2 allows to further classify precipitation intensities (Table 3). True-zero minutes (flag1=3) correspond to flag2=10. Value 11 assigns spurious precipitation with less than 20 particles with less than 5 bins occupied. The associated insignificant precipitation rates may result from very light precipitation or strong vibration of the instrument resulting in artificial signals. The decision is left to the user whether to consider or discard these values. Value 12 contains all precipitation minutes with a rate of 0.00 mm h^−1^. Values of 13 to 17 indicate increasing precipitation rates according to Table 3.

Additionally, we provide a physically-derived convective-stratiform precipitation index for rainfall. Modules for snow and mixed-phase precipitation are under development for future releases of the OceanRAIN dataset. Nevertheless, Rayleigh reflectivities are provided for snow and mixed phase but should be treated with care. Due to the unknown liquid-to-solid ratio, mixed-phase precipitation carries the largest uncertainty and should therefore be treated with care, as well. Precipitation minutes that fail the quality check are either set to true-zero precipitation or instrument malfunction. Rejection reasons include interference by wave water, riming, icing, birds and maintenance. However, the original values can be tracked in the OceanRAIN-W dataset using the number of particles, number of bins and the theoretical rain and snowfall values.

### Point-to-area representativeness of precipitation

The validation of satellite-derived precipitation estimates using surface-based point measurements of precipitation includes a number of challenges to be tackled. Loew *et al*.^65^ provide a comprehensive overview of these difficulties and ways how to address them properly. Resolution differences in space and time belong to the most important issues, constituting the long-standing point-to-area (p2a) problem. This problem influences both, the detection as well as the estimation of precipitation rates from point measurements within the satellite pixel area. However, the resulting representativeness error can be minimized by increasing the representativeness of point measurements such as OceanRAIN compared to an area as a satellite sensor sees it. To achieve this for OceanRAIN, Burdanowitz *et al*.^66^ conducted a synthetic study using weather radar data from the Rain In Cumulus clouds over the Ocean (RICO) campaign on Barbuda^99^. From the radar data, synthetic ship tracks within a synthetic area of a typical passive microwave satellite pixel were randomly matched to each other. This idealized study allows to isolate the representativeness error and to derive statistical adjustments for the along-track averaged precipitation rates with respect to the area-averaged precipitation rates to minimize the representativeness error. Both adjustments use parameters calculated from OceanRAIN, like the along-track precipitation event duration and the median-normalized along-track precipitation rate after the previous adjustment. The derived adjustment procedures from the purely synthetic radar study have been applied to OceanRAIN precipitation data to improve the evaluation of precipitation estimates from the HOAPS^23,28^ satellite climatology (Hamburg Ocean Atmosphere Parameters and fluxes from Satellite). The result has been compared in Burdanowitz^67^ using the Cloud Physical Properties (CPP) product^100^. A direct comparison in combination with a case study shows first, that the statistical adjustments perform well for most cases. Only widespread precipitation of uniform intensity needs no adjustment due to its spatially uniform precipitation distribution. Second, the p2a problem contributes more strongly to differences between HOAPS and OceanRAIN than the precipitation regime classified using precipitation area and intensity from CPP. The statistical adjustment of OceanRAIN is particularly crucial for rather coarsely resolved satellite estimates of 0.5° and above and marks an important step towards a more representative precipitation validation of satellite data over the ocean.

## Additional information

**How to cite this article**: Klepp, C. *et al*. OceanRAIN, a new in-situ shipboard global ocean surface-reference dataset of all water cycle components. *Sci. Data* 5:180122 doi: 10.1038/sdata.2018.122 (2018).

**Publisher’s note**: Springer Nature remains neutral with regard to jurisdictional claims in published maps and institutional affiliations.

## Supplementary Material



## Figures and Tables

**Table 1:** Overview of the OceanRAIN ship fleet, time period, oceans covered and sampling for all parameters and precipitation occurrence.

ship name	ship identifier	country	time period covered	main ocean basins covered	minutes in database	minutes with precipitation
RV Polarstern	DBLK	Germany	Jun2010—Oct2016	Atlantic	3,264,480	446,006
RV Meteor	DBBH	Germany	Mar2014—Mar2016	Atlantic	1,058,400	20,300
RV Maria S. Merian	DBBT	Germany	Oct2012—Jun2014	Atlantic	856,229	90,648
RV Sonne1	DFCG	Germany	Sep2012—Oct2012	Pacific	36,000	4,574
RV Sonne2	DBBE	Germany	Nov2014—Apr2017	Pacific	1,245.592	64,732
RV Investigator	VLMJ	Australia	Jan2016—Feb2017	Southern Ocean	303,144	54,814
RV Roger Revelle	KAOU	USA	Aug2016-Sep2016	Pacific	37,439	10,769
MS The World	C6RW4	Nassau	Jan2017-Feb2017	Southern Ocean	29,081	4,897
**sum of all ships**	**----**	**----**	**Jun2010—Apr2017**	**worldwide**	**6,830,365**	**696,740**
The data files are separated for each ship and contain the ship name and ship identifier						

**Table 2:** OceanRAIN flag1 convention for the ODM470 precipitation parameter.

Flag1	precipitation phase and ODM instrument condition
0	rainfall occurrence
1	snowfall occurrence
2	mixed-phase precipitation occurrence
3	true-zero value, no precipitation occurrence
4	inoperative instrument, no ODM data recorded
5	harbor time, no data recorded
9	missing value

**Table 3:** OceanRAIN flag2 convention for the ODM precipitation parameter.

Flag2	classification	information
10	true-zero value, no precipitation	No precipitation occurrence. Note, if allocated bins and numbers are not equal zero this minute was identified as an electronic artefact and the precipitation rate is set to zero.
11	spurious signals or extremely light precipitation	Precipitation occurrences with number of bins smaller to 5 and number of particles smaller to 20. The rates are insignificant or zero. Reasons for such signals be include real precipitation, vibration of the instrument or any kind of artifacts. It is left to the user to consider these minutes as being precipitation or not.
12	insignificant precipitation occurrence	Precipitation rates lower than 0.01 mm h^−1^ are set to 0.00 mm h^−1^. These are minutes with insignificant precipitation rates.
13	very light precipitation occurrence	Precipitation rates from 0.01 to 0.09 mm h^−1^. The values are below the threshold of what typical gauges are able to measure.
14	light precipitation occurrence	Precipitation rates from 0.1 to 0.99 mm h^−1^. The values are above the threshold of what typical gauges are able to measure.
15	moderate precipitation occurrence	Precipitation rates from 1.00 to 9.99 mm h^−1^ containing light to moderate precipitation events. The stratiform-convective flag can be additionally used to separate these minutes.
16	intense precipitation occurrence	Precipitation rates from 10.00 to 49.99 mm h^−1^ containing intense, convective precipitation.
17	extreme precipitation occurrence	Precipitation rates above 50 mm h^−1^ contain extreme convective precipitation events.
99	missing value	missing data.

**Table 4:** OceanRAIN metadata for instruments installation height and depth (m) for the derivation of the COARE Bulk Flux parameters and the evaporation.

ship name	wind speed height [m]	air temperature and relative humidity height [m]	water temperature depth [m]	precipitation height [m]
RV Polarstern	39.0	29.0	−5.0	39.0
RV Meteor	37.5	37.5	−2.5	37.5
RV Maria S Merian	30.8	20.2	−4.2	20.2
RV SonneI	21.6	21.6	−4.0	21.6
RV SonneII	34.0	27.0	−2.0	27.0
RV Investigator	22.1	31.4	−6.9	31.4
RV Roger Revelle	18.0	16.5	−0.05	10.0

**Table 5:** OceanRAIN-W Water Cycle Components. 73 parameters in temporally continuous 1min-resolution

# Parameter description OceanRAIN-W	NAME	Error value	Units	Data source	Format, Algorithm, Metadata, Citation
01 counter	COUNT	----------	[ ]	calculated	
02 date UTC	DATE	----------	UTC	NAV	DDMOYYYY
03 time UTC	TIME	----------	UTC	NAV	HHMM
04 date local	LDATE	----------	LT	calculated	DDMOYYYY
05 time local	LTIME	----------	LT	calculated	HHMM
06 minute of day UTC	MDAY	----------	[ ]	calculated	value range 1 to 1440
07 Julian date	JLD	----------	days	calculated	since 01JAN1994, 00 UTC
08 Unix epoch timestamp	USEC	----------	s	calculated	seconds since 01JAN1970, 00 UTC
09 latitude	LAT	-99.9999	deg	NAV	degree north from -90 to 90°
10 longitude	LON	-999.9999	deg	NAV	degree east from -180° to 180°
11 heading	HEAD	-99.9	deg	NAV	0° to 360°
12 air temperature	TAIR	-99.9	°C	MET	
13 dewpoint temperature	TDEW	-99.9	°C	calculated	
14 bulk water temperature	WATER	-99.9	°C	MET	
15 sea surface temperature	SST	-99.9	°C	calculated	Donlon et al.^90^
16 relative humidity	RH	-99	%	MET	
17 specific humidity at sea surface	QS	-9.9	g kg^−1^	calculated	Murphy and Coop^89^
18 specific air humidity	QA	-9.9	g kg^−1^	calculated	Murphy and Coop^89^
19 air pressure	MSLP	-999.9	hPa	MET	at instrument height
20 relative wind speed	UREL	-9.9	m s^−1^	MET	
21 relative wind direction	RELDIR	-99	deg	MET	
22 true wind speed	UTRUE	-9.9	m s^−1^	MET	
23 true wind direction	TRUEDIR	-99	deg	MET	
24 wind speed in 10 m height	U10	-9.9	m s^−1^	calculated	Tennekes^88^
25 global radiation	GLORAD	-999.9	W m^−2^	MET	
26 visibility	VIS	-9999	m	MET	
27 ceiling	CEIL	-99999	m	MET	
28 max gusts	UMAX	-99.9	m s^−1^	MET	
29 salinity	SAL	-99.99	PSU	MET	
30 drag transfer coefficient	CD	-99.9	[ ]	calculated	Fairall et al.^87^
31 latent heat transfer coefficient	CE	-99.9	[ ]	calculated	Fairall et al.^87^
32 sensible heat transfer coefficient	CH	-99.9	[ ]	calculated	Fairall et al.^87^
33 warm layer flag	WLF	3	[ ]	calculated	Fairall et al.^87^
34 sensible heat flux	SHF	-9999	W m^−2^	calculated	Fairall et al.^87^
35 latent heat flux	LHF	-9999	W m^−2^	calculated	Fairall et al.^87^
36 evaporation E	EVAP	-999	mm h^−1^	calculated	Fairall et al.^87^
37 freshwater budget E-P	BUDG	-999	mm h^−1^	calculated	difference of E and P
38 rain gauge precipitation rate	GAUGE	-99.99	mm h^−1^	MET	
39 ww present weather code	WW	-99	[ ]	SYN	human weather type observation
40 W1 past weather code	W1	-99	[ ]	SYN	human weather type observation
41 W2 past weather code	W2	-99	[ ]	SYN	human weather type observation
42 99th percentile particle diameter	PERC	-999.99	mm	calculated	
43 theoretical rain rate disdrometer	TRAIN	-99.99	mm h^−1^	calculated	#43 or #44 is identical to #52
44 theoretical snow rate disdrometer	TSNOW	-99.99	mm h^−1^	calculated	#43 or #44 is identical to #52
45 probability for rain	PRAIN	-999.99	[ ]	calculated	value range 0.00 to 1.00
46 probability for snow	PSNOW	-999.99	[ ]	calculated	value range 0.00 to 1.00
47 probability for mixed-phase	PMIX	-999.99	[ ]	calculated	value range 0.00 to 1.00
48 precipitation flag1	FLAG1	9	[ ]	calculated	precipitation type and status
49 precipitation flag2	FLAG2	99	[ ]	calculated	precipitation classification
50 number of bins	BINS	-99	[ ]	ODM	number of bins
51 number of particles	NUMS	-9999	[ ]	ODM	number of particles
52 ODM precipitation rate R	PRECIP	-99.99	mm h^−1^	calculated	according to #42-48
53 Rayleigh reflectivity Z	REFL	-99.99	mm^6^ m^−3^	calculated	
54 10 log R	DBR	-99.99	dBR	calculated	
55 10 log Z	DBZ	-99.99	dBZ	calculated	
56 relative wind speed	ODMREL	-88.88	m s^−1^	ODM	flag1= 4 or 5; missval= -99.99
57 reference voltage	UREF	-88.88	V	ODM	flag1= 4 or 5; missval= -99.99
58 convective=1 /stratiform=0 index	CONV	-9	[ ]	calculated	Thurai et al.^81^, No* < -1.65 * Dm + 6.35
59 Intercept of normalized gamma DSD	No*	-999.00	mm^−1^ m^−3^	calculated	Testud et al.^81^
60 mass-weighted mean diameter of normalized gamma DSD	Dm	-999.00	mm	calculated	Testud et al.^81^
61 shape parameter of normalized gamma DSD	mu	-999.00	[ ]	calculated	Testud et al.^81^
62 median volume diameter of normalized gamma DSD	D0	-999.00	mm	calculated	Testud et al.^81^
63 DSD mass spectrum standard deviation	sigmam	-999.00	mm	calculated	Williams et al.^83^
64 Intercept parameter of a standard gamma DSD	N0	-999.00	mm^−1^ m^−3^	calculated	Tokay and Short^82^
65 T-matrix simulation of C-band reflectivity from DSD	DBZ_C	-999.00	dBZ	calculated	pyTmatrix
66 T-matrix simulation of C-band differential reflectivity from DSD	ZDR_C	-999.00	dB	calculated	pyTmatrix
67 T-matrix simulation of C-band specific differential phase from DSD	KDP_C	-999.00	deg km^−1^	calculated	pyTmatrix
68 T-matrix simulation of Ku-band reflectivity from DSD	DBZ_Ku	-999.00	dBZ	calculated	pyTmatrix
69 T-matrix simulation of Ku-band differential reflectivity from DSD	ZDR_Ku	-999.00	dB	calculated	pyTmatrix
70 T-matrix simulation of Ku-band specific differential phase from DSD	KDP_Ku	-999.00	deg km^−1^	calculated	pyTmatrix
71 T-matrix simulation of Ka-band reflectivity from DSD	DBZ_Ka	-999.00	dBZ	calculated	pyTmatrix
72 T-matrix simulation of Ka-band differential reflectivity from DSD	ZDR_Ka	-999.00	dB	calculated	pyTmatrix
73 T-matrix simulation of Ka-band specific differential phase from DSD	KDP_Ka	-999.00	deg km^−1^	calculated	pyTmatrix
The data source discriminates between measured (NAV, MET, ODM), observed (SYN) and calculated parameters (calculated).					

**Table 6:** OceanRAIN-M number concentration PSDs (m^−3^ mm^−1^) and OceanRAIN-R raw PSDs along with and precipitation microphysics and radar-related parameters.

# Parameter description OceanRAIN-M and OceanRAIN-R	NAME	Error value	Units	Data source	Format, Algorithm, Metadata, Citation
01 counter	COUNT	----------	[ ]	calculated	
02 date UTC	DATE	----------	UTC	NAV	DDMOYYYY
03 time UTC	TIME	----------	UTC	NAV	HHMM
04 minute of day UTC	MDAY	----------	[ ]	calculated	value range 1 to 1440
05 Julian date	JLD	----------	days	calculated	since 01JAN1994, 00 UTC
06 Unix epoch timestamp	USEC	----------	s	calculated	seconds since 01JAN1970, 00 UTC
07 latitude	LAT	-99.9999	deg	NAV	degree north from -90 to 90°
08 longitude	LON	-999.9999	deg	NAV	degree east from -180° to 180°
09 probability for rain	PRAIN	-999.99	[ ]	calculated	value range 0.00 to 1.00
10 probability for snow	PSNOW	-999.99	[ ]	calculated	value range 0.00 to 1.00
11 probability for mixed-phase	PMIX	-999.99	[ ]	calculated	value range 0.00 to 1.00
12 precipitation flag1	FLAG1	9	[ ]	calculated	precipitation type and status
13 precipitation flag2	FLAG2	99	[ ]	calculated	precipitation classification
14 number of bins	BINS	-99	[ ]	ODM	number of bins
15 number of particles	NUMS	-9999	[ ]	ODM	number of particles
16 ODM precipitation rate R	PRECIP	-99.99	mm h^−1^	calculated	according to #42-48
17 Rayleigh reflectivity Z	REFL	-99.99	mm^6^ m^−3^	calculated	
18 10 log R	DBR	-99.99	dBR	calculated	
19 10 log Z	DBZ	-99.99	dBZ	calculated	
20 relative wind speed	ODMREL	-88.88	m s^−1^	ODM	flag1= 4 or 5; missval= -99.99
21 reference voltage	UREF	-88.88	V	ODM	flag1= 4 or 5; missval= -99.99
22 convective=1 /stratiform=0 index	CONV	-9	[ ]	calculated	Thurai et al.^81^, No* < -1.65 * Dm + 6.35
23 Intercept of normalized gamma DSD	No*	-999.00	mm^−1^ m^−3^	calculated	Testud et al.^81^
24 mass-weighted mean diameter of normalized gamma DSD	Dm	-999.00	mm	calculated	Testud et al.^81^
25 shape parameter of normalized gamma DSD	mu	-999.00	[ ]	calculated	Testud et al.^81^
26 median volume diameter of normalized gamma DSD	D0	-999.00	mm	calculated	Testud et al.^81^
27 DSD mass spectrum standard deviation	sigmam	-999.00	mm	calculated	Williams et al.^83^
28 Intercept parameter of a standard gamma DSD	N0	-999.00	mm^−1^ m^−3^	calculated	Tokay and Short^82^
29 T-matrix simulation of C-band reflectivity from DSD	DBZ_C	-999.00	dBZ	calculated	pyTmatrix
30 T-matrix simulation of C-band differential reflectivity from DSD	ZDR_C	-999.00	dB	calculated	pyTmatrix
31 T-matrix simulation of C-band specific differential phase from DSD	KDP_C	-999.00	deg km^−1^	calculated	pyTmatrix
32 T-matrix simulation of Ku-band reflectivity from DSD	DBZ_Ku	-999.00	dBZ	calculated	pyTmatrix
33 T-matrix simulation of Ku-band differential reflectivity from DSD	ZDR_Ku	-999.00	dB	calculated	pyTmatrix
34 T-matrix simulation of Ku-band specific differential phase from DSD	KDP_Ku	-999.00	deg km^−1^	calculated	pyTmatrix
35 T-matrix simulation of Ka-band reflectivity from DSD	DBZ_Ka	-999.00	dBZ	calculated	pyTmatrix
36 T-matrix simulation of Ka-band differential reflectivity from DSD	ZDR_Ka	-999.00	dB	calculated	pyTmatrix
37 T-matrix simulation of Ka-band specific differential phase from DSD	KDP_Ka	-999.00	deg km^−1^	calculated	pyTmatrix
38-165: 128-times number concentration PSD	PSD	----------	m^−3^ mm^−1^	calculated	OceanRAIN-M files
38-165: 128-times raw particle count PSD	RAW	----------	n	ODM	OceanRAIN-R files
Both files contain 37 parameters in temporally discontinuous 1min-resolution for precipitation events only. The data source discriminates between measured (NAV, MET, ODM), observed (SYN) and calculated parameters (calculated). Note, that column 38-165 store the 128 values for the PSDs. The first lines in the OceanRAIN-M files contain a header providing the log-scale 128 bin size centers in mm.					
